# Measured by the oxygen uptake in the field, the work of refuse collectors is particularly hard work: Are the limit values for physical endurance workload too low?

**DOI:** 10.1007/s00420-015-1064-8

**Published:** 2015-06-19

**Authors:** Alexandra M. Preisser, Linfei Zhou, Marcial Velasco Garrido, Volker Harth

**Affiliations:** Institute for Occupational and Maritime Medicine (ZfAM), University Medical Center Hamburg-Eppendorf, Seewartenstrasse 10, 20459 Hamburg, Germany

**Keywords:** Oxygen uptake, Physically heavy work, Mobile spiroergometry, Relative heart rate, Waste collectors, Endurance work

## Abstract

**Purpose:**

Collecting waste is regarded as a benchmark for “particularly heavy” work. This study aims to determine and compare the workload of refuse workers in the field. We examined heart rate (HR) and oxygen uptake as parameters of workload during their daily work.

**Methods:**

Sixty-five refuse collectors from three task-specific groups (residual and organic waste collection, and street sweeping) of the municipal sanitation department in Hamburg, Germany, were included. Performance was determined by cardiopulmonary exercise testing (CPX) under laboratory conditions. Additionally, the oxygen uptake (VO_2_) and HR under field conditions (1-h morning shift) were recorded with a portable spiroergometry system and a pulse belt.

**Results:**

There was a substantial correlation of both absolute HR and VO_2_ during CPX [HR/VO_2_*R* 0.89 (SD 0.07)] as well as during field measurement [*R* 0.78 (0.19)]. Compared to reference limits for heavy work, 44 % of the total sample had shift values above 30 % heart rate reserve (HR_R_); 34 % of the individuals had mean HR during work (HR_sh_) values that were above the HR corresponding to 30 % of individual maximum oxygen uptake (VO_2,max_). All individuals had a mean oxygen uptake (VO_2,1h_) above 30 % of VO_2,max_.

**Conclusion:**

HR as well as the measurement of VO_2_ can be valuable tools for investigating physiological workload, not only under laboratory conditions but also under normal working conditions in the field. Both in terms of absolute and relative HR and oxygen consumption, employment as a refuse collector should be classified in the upper range of defined heavy work. The limit of heavy work at about 33 % of the individual maximum load at continuous work should be reviewed.

## Introduction

The organized collecting of waste is essential for a functioning community; however, there is no explicit job qualification connected with, and the work of garbage collectors receives little scientific attention. Collecting waste is described as physically demanding work and as being the cause of various physical disorders with respiratory, gastrointestinal, and musculoskeletal symptoms (Kuijer and Frings-Dresen 2004; Kuijer et al. 2010). This work is regarded as a benchmark for “particularly heavy” work. The definition of “heavy work” is based so far only on the assumption that the endurance limit is 30 % respectively 33 % of the maximum load capacity, taking into account load peaks, manual work, and harmful temperatures (Ilmarinen et al. 1991; Rutenfranz et al. 1976). The determination of an “upper limit” is essential for defining the “reasonableness” of a work—in the sense of the absence of excessive risks to health. There are presently also no indications, showing how the physical performance is with this heavy work with increasing age. An assessment is required in order to meet the challenges of demographic change in industrialized countries. Only few studies have investigated in detail the refuse collectors in different countries with different tasks. Up to now, the heart rate (HR) is used as an indirect indicator of the physiological workload, for example in the Netherlands (Kemper et al. 1990), Japan (Tsujimura et al. 2012), and Brazil (Anjos et al. 2007). The oxygen uptake (VO_2_), as a direct measure of the metabolic processes, however, was mostly estimated via HR in these groups. So far, the VO_2_ of refuse collectors was determined only once by means of simulation in the laboratory (Kemper et al. 1990; Frings-Dresen et al. 1995). The relation between HR and VO_2_ has not yet been specified under field conditions. This may be due to the fact that the measurement of oxygen uptake with a breathing mask for outdoor work in this occupation group is technically particularly challenging. In our view, however, the conclusion of HR on VO_2,max_ requires a review. To our knowledge, there are no recent studies with refuse collectors, who were investigated during their daily work with portable spiroergometry to determine the real oxygen uptake.

This paper is based on a study about the physiological workload of 65 employees from three task-specific groups [residual waste collection (RWC), organic waste collection (OWC), and street cleaning (SC)] of a municipal sanitation department in Germany. Our aim was to categorize the respective workload of these professions under real working conditions as a contribution to the development of a classification of workload in occupational health research. To evaluate the methods in the field of measurement, we also conducted comparisons of the methods of workload measurement. For this purpose, HR and oxygen uptake were determined in field measurements. For comparison, we measured the oxygen uptake by a stationary cycle cardiopulmonary exercise test (CPX).

## Methods

The study group consisted of 65 subjects (62 males and 3 females), aged between 25 and 60, all employees in the municipal sanitation department in Hamburg, Germany. All participants volunteered and were granted compensatory time off by the employer. Before the start of the investigations, there was no selection of participants. The anthropometric characteristics of the subjects (Table 1) are representative in age and sex of the 1544 employees [46.5 (SD 8.6) years; 98 % male) working in refuse collecting in this sanitation department. The examined employees were subdivided by their occupational tasks into three groups: RWC (*n* = 35), OWC (12), and SC (18). These jobs are mainly performed by male employees, although there are a few females in street sweeping in Hamburg. There were three women in the last group. The Declaration of Helsinki has been adequately addressed, and written informed consent was obtained from all participants. The study was approved by the Ethics Committee of the Hamburg Medical Association (register number PV4524).

**Table 1:** Characteristics of study participants

	*N*	Female	Age (years)		Height (m)		Weight (kg)		BMI (kg/m^2^)	
			Mean	SD	Mean	SD	Mean	SD	Mean	SD
All	65	3	45.6	8.3	177.7	7.6	89.7	14.7	28.3	3.8
Male	62		45.5	8.4	177.0^a^	8.3	88.7^a^	15.3	28.2	3.9
Female	3	3	43.1	11.5	162.0	9.6	65.7	7.4	25.2	4.3
RWC	35	–	47.3^b^	7.0	179.1	7.4	92.5	15.5	28.8	4.0
OWC	12	–	46.7	8.3	177.8	5.3	94.3	12.8	30.0	4.0
SC	18	3	41.6	9.7	175.0	9.0	81.2^b,c^	11.1	26.5^b,c^	2.8
All_field_	13	2	49.7	6.7	174.8	9.3	87.1	15.9	28.3	4.0
RWC_field_	5	–	51.1	4.0	178.6	11.5	88.6	12.5	27.6	1.2
OWC_field_	3	–	50.4	6.3	175.7	3.8	103.0	14.5	33.4^d^	4.9
SC_field_	5	2	47.8	9.6	170.6	9.0	76.0^e^	12.3	26.0	2.8

*SD* Standard deviation, *BMI* body mass index, *RWC* residual waste collectors, *OWC* organic waste collectors, *SC* street cleaners, *All*_*field*_ subjects submitted to field measurement with portable spiroergometric system

Founded significant differences between ^a^male/female; ^b^ RWC/SC; ^c^ OWC/SC, ^d^ OWC_field_/all_field_, and ^e^ SC_field_/all_field_. The first four differences can be explained by the inclusion of women in the SC group, the latter not

Elements of investigation were specific questioning and physical examination (regarding occupation, symptoms, and disorders according to body functions). Furthermore, spirometry, body plethysmography (MasterScreen™ Body by JAEGER™/CareFusion, Hoechberg, Germany), and CPX were performed with 61 subjects. Four persons were excluded due to cardiorespiratory risk factors.

Spirometry represents a measure of forced one-second capacity and vital capacity (FEV_1_, FVC) performed according to the criteria of the American Thoracic Society (1995) with the calculation of FEV_1_/FVC. In addition, body plethysmography determines the airways resistance as well as intrathoracic gas volume.

CPX was performed according to the recommendations of the German Society of Pneumology (Meyer et al. 2013) with 12-lead ECG monitoring on an electronically braked computer-controlled cycle ergometer (ergoselect 200p/Ergoline Bitz, Germany) with a continuous increase in the load. This ramp-like protocol enables a precise determination of maximal aerobic and power output and the ventilatory threshold (VT) (Binder et al. 2008; Meyer et al. 2005). Performance and VO_2_ and carbon dioxide outputs (VCO_2_) were measured continuously (Oxycon Pro™ by JAEGER™/CareFusion, Hoechberg, Germany).

CPX was preceded by 2 min of sitting at rest. After a warm-up period of 2 min with an external workload of 25 W, the exercise followed with an increase of 15–25 W/min (Meyer et al. 2013) depending on the individual fitness level. Subjects were verbally encouraged until they could no longer sustain the required crank frequency of 60–70 rpm. Maximum oxygen uptake (VO_2_,_max_) was calculated as the average of the highest eight consecutive breaths in the final minute of exercise. The standard equations by Hansen et al. (1984), Reiterer (1975), and Wasserman et al. (2004) for VO_2,max_ and maximal wattage (*P*_max_) were used for assessment. The VT corresponds to the first VT; it was determined with a combination of VCO_2_/VO_2_ slope and increase in minute ventilation (VE) relative to oxygen consumption (VE/VO_2_), ventilatory equivalent named. This first VT is defined by the increase in VE/VO_2_ without a concurrent increase in VE/VCO_2_ (Binder et al. 2008; Westhoff et al. 2013).

**Fig. 1:**
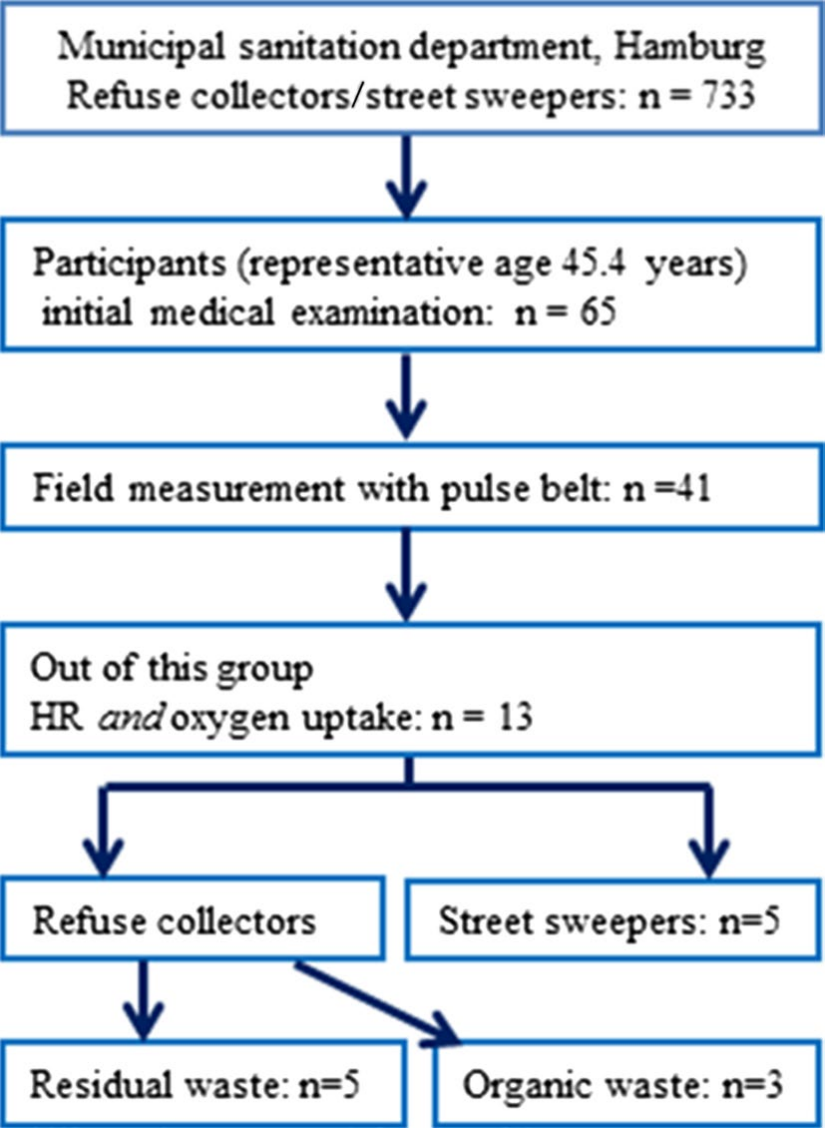
Flowchart of the measurements

**Fig. 2:**
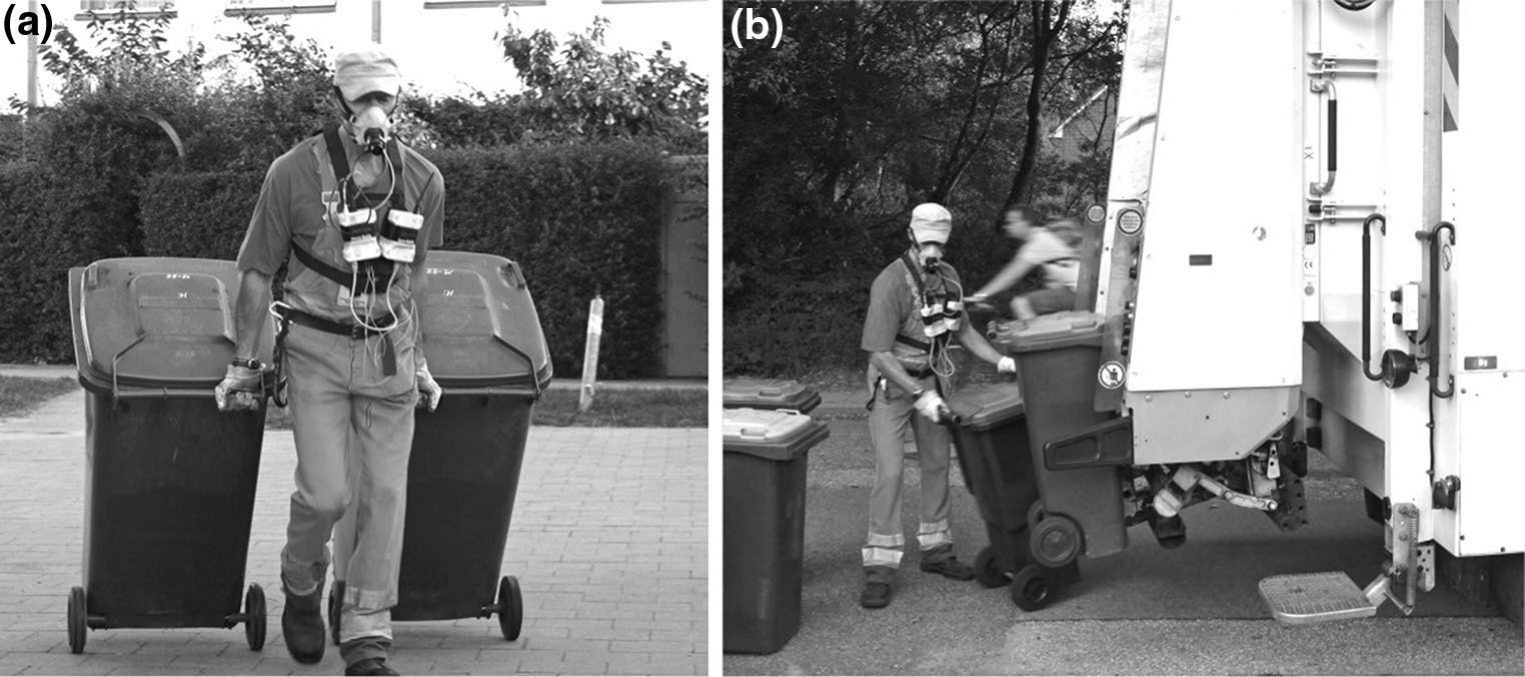
**a**, **b** Refuse collector with spiroergo mask, equipment, and garbage cans

Forty-one subjects were studied while working with long-term HR measurements (T31 coded transmitter, Polar Electro, Buettelborn, Germany) during a work shift (mean 6.7 h). From this group, 20 subjects (18 males and 2 females) were also connected to mobile CPX (Oxycon Mobile by JAEGER™/CareFusion, Hoechberg, Germany) and to the HR monitoring system for an average of 1.3 h to measure the correlation between HR and oxygen uptake under field conditions (HR_field_, VO_2,field_) (Fig. 1). The field measurement was started before the truck left the depot and thus recorded approximately 30 min of driving plus 1 h of sustained work. The actual HR and oxygen uptake under task-specific work were recorded during the following 1 h of continuous work (HR_1h_, VO_2,1h_). Part of the work of the garbage collectors is transporting two-wheeled waste containers (120 l volume) of houses and cellars and the shift of large four-wheeled waste containers (240 l) of storerooms. The path length of an entire work day was estimated with a pedometer and was about 7–10 km. All waste containers were emptied machine-supported into the truck (Fig. 2a, b, photographs with waste worker, spiroergo mask, and garbage cans). Occasional waste bags were towed. SC consisted of sweeping waste and leaves, sometimes wet leaves, as well as picking up trash. Due to malfunction of the measuring instruments, refusal, and changes in the organization, valid data were obtained for only 13 of 20 subjects.

Ahead of the gas exchange measurements in the field via face mask, the mobile CPX unit was volume and gas calibrated. HR and oxygen uptake were both depicted in absolute values and relative to individuals’ maximum values (%HR_max_, %VO_2,max_) and individuals’ values at the VT (%HR_VT_,  %VO_2,VT_). The difference to maximum values as “reserve values” (%HR_R_,  %VO_2,R_) was defined as: (HR_work_ − HR_rest_)/(HR_max_ − HR_rest_) × 100 %, and (VO_2,work_ − VO_2,rest_)/(VO_2,max_ − VO_2,rest_) × 100 %, respectively. The HR and VO_2_ at rest (HR_rest_,VO_2,rest_) were calculated from the mean values in the first 2 min of the exercise test and the previously measured resting value.

## Statistics

Data are presented as means and standard deviations (SD). To assess the equivalence of linear regression, mean values for Pearson correlation (*R*), intercept, and slope were determined for each individual. Student’s *t* test and Wilcoxon test were used to determine whether the mean intercepts and slopes differed from 0 to 1, respectively, and to verify differences between sample characteristics and differences from reference limits. All calculations were performed using IBM SPSS Statistics 22. For all statistical analyses, the null hypothesis was rejected at a probability of *p* < 0.05.

## Results

The 65 subjects of the study group showed only low differences in age and in body mass within the total sample for RWC, OWC, and SC, respectively (Table 1). All 61 subjects who could participate in the CPX had normal ECG readings and took no HR-affecting drugs. On the basis of spirometry and body plethysmography, obstructive lung disease (FEV_1_/FVC < 70 %) was observed in 21.5 % of subjects. All workers diagnosed with pulmonary disorders were active or former smokers (35.4 and 43.1 % of total sample, respectively).

The results from HR measurement at work (HR_sh_) of 41 subjects with an average work shift time of 6.7 h are shown in Table 2. Mean values of the total sample were 100.2 b/min and 27.9 % HR_R_, respectively. The HR_sh_ values relative to individuals’ HR_max_ and to HR_VT_ (%HR_max_, %HR_VT_) determined in the laboratory CPX showed that the OWC had the highest strain compared with the three subgroups (data not shown in detail).

**Table 2:** Mean heart rate during a work shift (HR_sh_) of 6.7 h of *n* = 41, percentage of maximal heart rate (%HR_max_), and heart rate at the ventilatory threshold (%HR_VT_) from CPX (values relative to heart rate reserve (%HR_R_))

	*N*	Female	Mean HR_sh_ (b/min)		HR_sh,max_ (b/min)		%HR_max_		%HR_VT_		%HR_R,sh_	
			Mean	SD	Mean	SD	Mean	SD	Mean	SD	Mean	SD
All	41	3	100.2	11.9	153.7	28.2	63.1	9.2	79.4	12.9	27.9	14.2
RWC	18	–	93.4^a,b^	11.8	150.6	30.5	58.3^a,b^	8.6	75.8	16.2	25.3	13.7
OWC	9	–	107.8	7.5	152.8	22.4	68.8	8.9	85.2	8.9	26.1	14.8
SC	14	3	103.9	9.9	158.2	29.8	65.7	7.4	80.1	9.1	32.5	14.2

*RWC* Residual waste collectors, *OWC* organic waste collectors, *SC* street cleaners

^a^Significant difference RWC/OWC

^b^Significant difference RWC/SC

HR recorded during one representative work hour (Table 3) showed a slightly higher mean HR_1h_ of 109.2 b/min and 45.1 % HR_R_, respectively, for the 13 subjects (for whom also the oxygen uptake was measured) than in the measurement over the whole work shift of the total sample. There were no significant differences between the groups OWC, RWC, and SC for 1 h of measurement. Mean HR values during 1 h as well as during a work shift were close to the HR at VT. Between HR_1h_ and HR_sh_ of these 13 subjects, there was a mean correlation coefficient of R 0.64. The regression of HR_1h_ was slightly but significantly (*p* < 0.05) higher than HR_sh_ by 10.6 b/min. During the one representative working hour (Table 3), the group mean achieved an oxygen uptake (VO_2,1h_) of 1103 ml/min. Here too, mean VO_2_ was close to VO_2,VT_. The groups did not differ significantly.

**Table 3:** Average of heart rate (HR_1h_) and oxygen uptake (VO_2,1h_) during 1 h of work

	*N*	HR_1h_ (b/min)		%HR_R,1h_		% HR_max_		%HR_VT_		VO_2,1h_ (ml)		%VO_2,max_		%VO_2,VT_	
		Mean	SD	Mean	SD	Mean	SD	Mean	SD	Mean	SD	Mean	SD	Mean	SD
All_field_^a^	13	109.2	12.5	45.1	18.9	71.1	11.5	86.7	17.2	1103	237.3	45.7	9.3	60.0	14.3
RWC_field_	5	106.4	15.0	38.3	20.3	66.6	9.8	84.5	22.1	1160	137.0	42.9	5.8	57.8	15.1
OWC_field_	3	106.5	9.1	32.7	12.7	67.2	13.5	82.4	14.3	1286	80.2	50.8	7.2	69.1	13.3
SC_field_^a^	5	113.7	12.7	59.3	12.6	78.0	10.7	91.6	15.8	935	287.1	45.2	13.0	56.7	14.5

HR_1h_ and VO_2,1h_ relative to maximal heart rate and maximal oxygen uptake during CPX (%HR_max_; %VO_2,max_). HR_1h_ and VO_2,1h_ relative to heart rate and oxygen uptake at the ventilatory threshold (%HR_VT_; %VO_2_,_VT_). And values relative to heart rate reserve (%HR_R_)

*RWC* Residual waste collection, *OWC* organic waste collection, *SC* street cleaning, *All*_*field*_ subjects submitted to field measurement with portable spiroergometric system

The results of CPX with measurement of VO_2_ of all 61 participants and of the 13 subjects with field measured data are depicted in Tables 4 and 5. The three subgroups RWC, OWC, and SC do not differ significantly in this test with respect to *P*_max_, VO_2,max_, and HR_max_ (data not shown). The spiroergometric field measurements’ sample of 13 subjects did not differ significantly from the whole group. A relationship between the maximal values from *P*_max_ and VO_2,max_ could be observed with mean correlation coefficient (*R*) of 0.88; HR_max_ was weakly correlated with age (*R* 0.45). Therefore, older participants showed surprisingly a slight increase in HR_max_ with age (data not shown in detail). The individuals reached values close to age-predicted values with 95.6 % (SD 18.2) VO_2,max_/VO_2,pred_, and 90.8 % (SD 7.4) HR_max_/HR_pred_, (Hansen et al. 1984; Reiterer 1975; Wasserman et al. 2004).

**Table 4:** Results of CPX tests

	*N*	Female	*P*_max_ (W)		*P*_max_ (W/kg)		VO_2,max_ (ml)		VO_2,max_ (ml/kg)		HR_max_ (b/min)		RER_max_	
			Mean	SD	Mean	SD	Mean	SD	Mean	SD	Mean	SD	Mean	SD
All	61^a^	3	192.1	39.2	2.1	0.5	2623	571	29.2	6.4	158.9	14.3	1.27	0.12
All_field_	13	2	184.5	46.9	2.1	0.5	2458	562	28.4	5.5	155.2	14.1	1.26	0.13
RWC_field_	5	–	200.0	17.0	2.3	0.2	2739	398	31.0	2.2	160.0	4.8	1.25	0.15
OWC_field_	3	–	180.0	56.3	1.8	0.6	2562	372	25.2	4.6	162.0	27.2	1.22	0.28
SC_field_	5	2	171.6	64.8	2.2	0.7	2113	682	27.7	7.8	146.2	6.4	1.28	0.06

^a^The difference to Table 1 can be explained by the exclusion of four people due to cardiac disease or medication

**Table 5:** Heart rate (HR_VT_), power output (*P*_VT_), and oxygen uptake (VO_2,VT_) at ventilatory threshold (VT) from CPX tests [relative to maximal values from CPX (%*P*_max_, % VO_2,max_, %HR_max_)]

	*N*	Female	*P* at VT (W)		VO_2_ at VT (ml)		HR at VT (b/min)		%*P*_max_		%VO_2,max_		%HR_max_	
			Mean	SD	Mean	SD	Mean	SD	Mean	SD	Mean	SD	Mean	SD
All	61	3	129.7	43.6	1843	498	125.1	15.3	66.9	16.3	70.3	11.8	79.0	9.3
All_field_	13	2	130.0	49.5	1914	549	128.4	15.8	69.2	12.6	77.1	8.0	82.9	8.4
RWC_field_	5	–	143.0	43.1	2109	514	129.6	20.1	70.5	17.0	76.0	9.3	81.0	12.7
OWC_field_	3	–	115.0	43.6	1908	400	131.3	19.3	63.1	4.4	74.0	4.5	81.3	2.4
SC_field_	5	2	126.0	64.5	1723	684	125.4	11.7	71.6	12.0	80.0	8.6	85.7	5.9

*RWC* Residual waste collectors, *OWC* organic waste collectors, *SC* street cleaners, *All*_*field*_ subjects submitted to field measurement with portable spiroergometric system

**Fig. 3:**
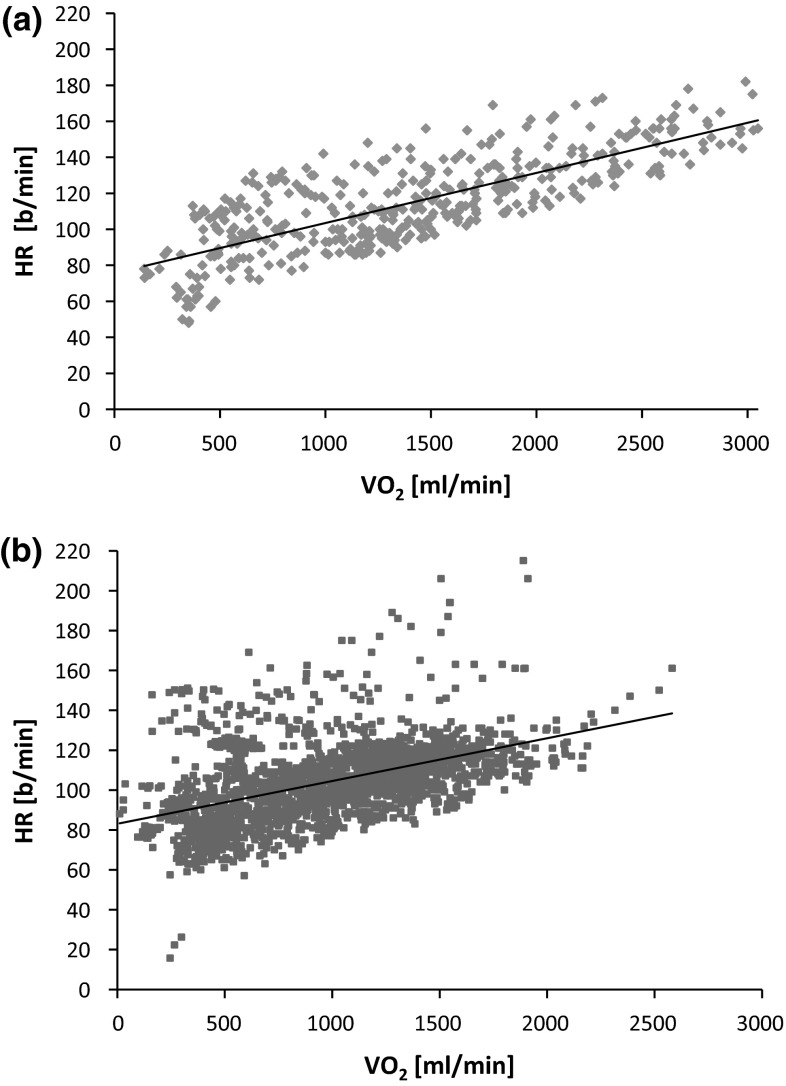
Heart rate (HR) and oxygen uptake (VO_2_) during CPX with increasing workload by 15–25 W/min (**a**) and during spiroergometric field measurement at work (**b**), for 13 subjects. **a** HR (b/min) = 0.03 × VO_2_ (ml/min) + 70.95 (drawn *trendline*); *R* ^2^ = 0.80; *n* = 426; *p* < 0.001. **b**. HR (b/min) = 0.03 × VO_2_ (ml/min) + 76.2 (drawn *trendline*); *R* ^2^ = 0.65; *n* = 2191; *p* < 0.001

**Fig. 4:**
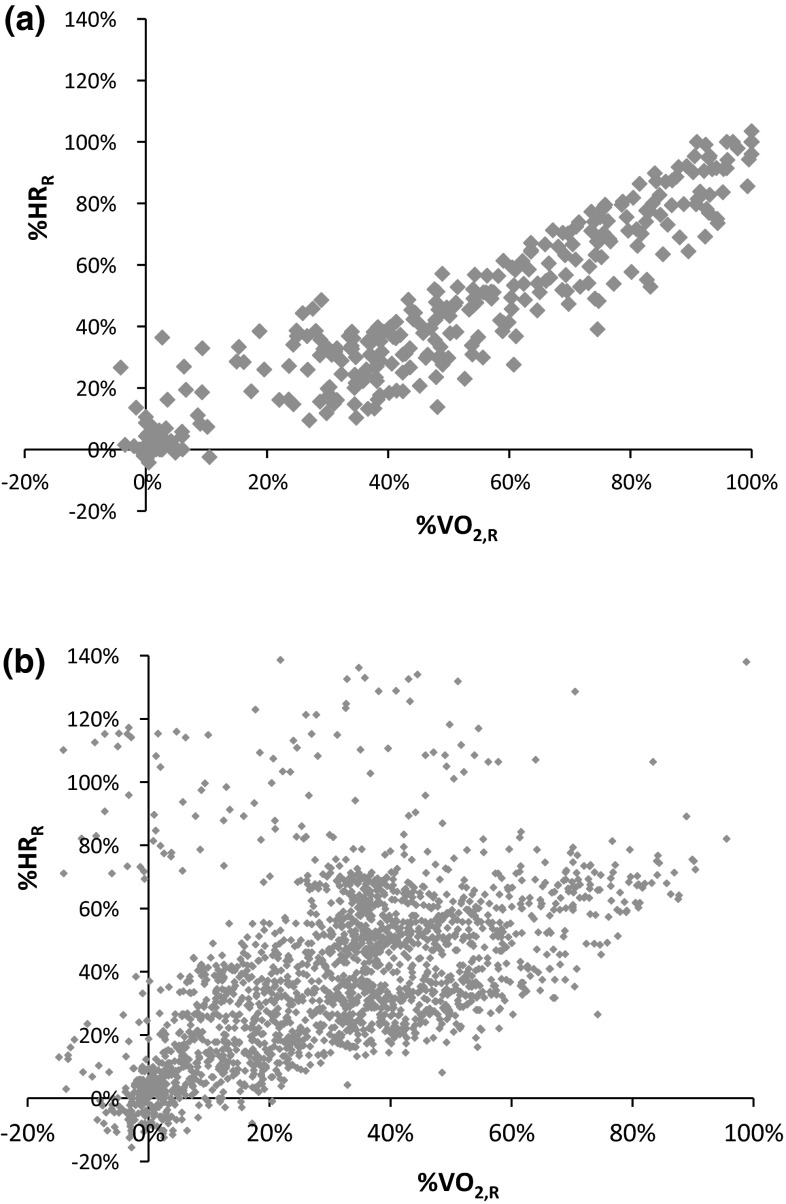
Heart rate as a percentage of heart rate reserve (%HR_R_) in relation to oxygen uptake as a percentage of oxygen uptake reserve (%VO_2,R_), determined during CPX (**a**) and field measurement (**b**), for 13 subjects. **a** %HR_R_ = 0.925 × %VO_2,R_ (ml/min) − 0.017; *R* ^2^ = 0.93; *n* = 325; *p* < 0.001. **b** %HR_R_ = 0.783 × %VO_2,R_ (ml/min) + 0.130; *R* ^2^ = 0.68; *n* = 2146; *p* < 0.001

The linear regression analysis was accomplished to study the relationship between HR and VO_2_ for CPX and field measurement. Data of HR and oxygen uptake during CPX create an individual linear heart/oxygen uptake relationship and a substantial correlation (mean *R* 0.89, *p* < 0.001). There was also a linear regression, with a mean correlation coefficient of *R* 0.78 (*p* < 0.001) between HR_field_ and VO_2,field_. The equations obtained here were nearly the same; both regressions for CPX and field measurement are shown in Fig. 3a, b. The correlation between %HR_R_ and %VO_2,R_ during CPX was high (*R* 0.96). The correlation during field measurement was similar, albeit lower, (*R* 0.78, both *p* < 0.001) (Fig. 4a, b).

**Fig. 5:**
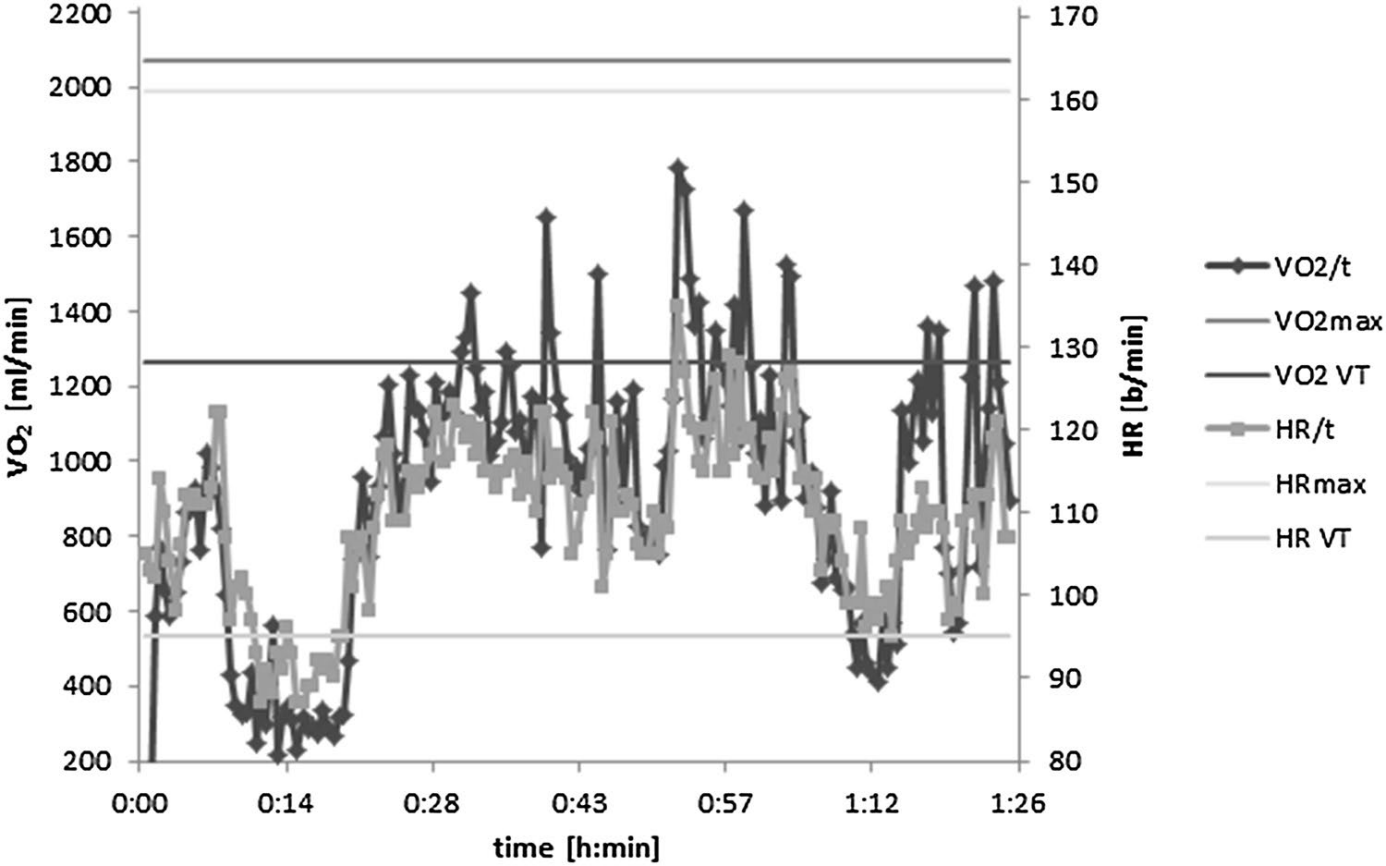
Case report: heart rate and oxygen uptake during field measurement (HR_field_,VO_2,field_) of one subject. Individual maximal heart rate, maximal oxygen uptake (HR_max_, VO_2,max_), and the values at ventilatory threshold (HR_VT_, VO_2,VT_) are also shown

During the field measurement of continuous HR_field_ and VO_2,field_, a simultaneous increase and decrease in HR and oxygen uptake could be observed in each individual. In Fig. 5, a typical example is given of one subject.

## Discussion

### Relationship of HR to VO_2_ to determine the validity of measuring methods in the field

Heart rate increases linearly as a function of workload intensity and is closely related to oxygen uptake (Arts and Kuipers 1994; Gastinger et al. 2010). Nevertheless, the value of the HR/VO_2_ relationship can vary between individuals due to metabolic stress or physical training level and therefore should be ascertained individually (Skinner et al. 2003). Similarly, interindividual differences are observed when CPX and field measurements during work are compared. To determine the physiological workload of physically demanding work, we investigated the relation between HR and oxygen uptake under field conditions. We could demonstrate that the HR/VO_2_ relationship was linear not just during the incremental cycle exercise test (CPX) but also in their usual working environment with climatic and other factors. Nevertheless, the range of the correlation coefficients shows that HR is more strongly correlated to VO_2_ during CPX (*R* 0.89, *p* < 0.001) than during field measurement (*R* 0.78, *p* < 0.001) (Fig. 3a, b). Yet, there is a significant correlation between HR and VO_2_ in the field measurement, and furthermore, a congruent increasing and decreasing profile could be demonstrated (Fig. 5).

Due to different proportions between HR and VO_2_, a method based on heart rate reserve (%HR_R_) and VO_2_ reserve (%VO_2,R_) is widely used for the comparison of relative values. Swain and Leutholtz (1997) and recent studies by Lounana et al. (2007) have shown that %HR_R_ data at group level are consistent with %VO_2,R_. We aimed to find out whether this correlation can also be validated for our group in CPX and especially in spiroergometric field measurement under working conditions. For the incremental exercise testing, we can confirm a substantial correlation of %HR_R_ and %VO_2,R_ with *R* 0.96 (*p* < 0.01) (Fig. 4a). For spiroergometric field measurement, we found a lower correlation of *R* 0.78 (*p* < 0.001). Both regressions show that %HR_R_ does not overestimate %VO_2,R_ as their intercepts are close to 0 (Fig. 4b). Possible reasons for lower field correlations between HR and VO_2_ could be malfunctioning in gathering the individual values, different load shapes, and the varyingly high intensity of physical strain of muscle groups with differing efficiency. During work as a refuse collector, especially arm work is performed, while the incremental cycle exercise consists mostly of legwork. A better equivalence between %HR_R_ and %VO_2,R_ for legwork than for arm work has been described by (Rotstein and Meckel 2000). Additionally, HR can be impaired by further factors, such as temperature, emotion, and physical fitness status (Achten and Jeukendrup 2003). We nevertheless could demonstrate an equivalence between absolute values of HR and VO_2_, and equally in relative calculations to HR_R_ and VO_2,R_ in dynamic work, even if it was measured in the field.

### Fitness and workload capacity evaluated by various thresholds and aspects

Because the VT reflects the workload threshold beyond which endurance exercise will not lead to anaerobic metabolism, it can therefore be regarded as the upper limit of intensity during the endurance performance (Binder et al. 2008). The present study showed a high endurance performance for the entire sample during 1 h of work and also during the whole work shift, depending on the HR measurement with a mean of 86.7 % HR_VT_ and 79.4 % HR_VT_, respectively. The percentage of VO_2_ during 1 h of work in percentage of VO_2,VT_ was likewise, but lower, with a mean of 60 % VO_2,VT_. In Fig. 5, which shows a representative measurement from the field tests, the subject’s HR well exceeded most of the time the individual HR_VT_. Similarly, VO_2,VT_ was exceeded several times. For individual values relative to the VT (%HR_VT_, %VO_2,VT_), our data show that %HR_VT_ may overestimate the real workload; %VO_2,VT_ seems to be more realistic (see Table 5). Furthermore, VT not only differs between individuals but also varies depending on the state of training and the type of exercise protocol (Faude et al. 2009). Therefore, the question arises whether %HR_VT_ is comparable to %VO_2,VT_. We would recommend to determine VT and likewise the HR_VT_ and VO_2,VT_, by CPX in the laboratory. This will enable an accurate estimate of %VO_2,VT_ during the field measurement.

In our sample, the CPX results are close to the individual predicted and age-dependent values (Table 2). Kroidl et al. (2014) have described the requirements for high, normal, and pathological endurance performance, based on values at VT > 80 %, around 60 %, and <40 % of maximal values, respectively. In comparison, our subjects also reached performance levels in the upper range of normal endurance (Table 2). In the present study, workers show normal ranges of individual fitness. Long work periods with a high level of physical activity did not lead to an increase in maximal oxygen uptake, and only slightly better endurance performance was observed in them. This seems to be compatible with results from previous studies which also investigated workers with heavy workload (Ilmarinen et al. 1991; Søgaard et al. 1996).

It is commonly suggested that 33–40 % of the individual’s VO_2,max_ should be the capable workload for 8 h of physical work (Åstrand et al. 2003; Ilmarinen 1992). But %VO_2,max_ depends on the type of exercise performed. According to Kemper et al. (1990), the acceptable limit for refuse collecting work in particular, which mainly consists of arm work combined with legwork, should be at 30 % VO_2,max_ for an 8-h shift. To describe the exercise intensity in our sample, we took HR_sh_ at a given %VO_2,max_. This method is according to Skinner et al. (2003); they have demonstrated that once VO_2,max_ and the relationship among HR and VO_2_ are known, the corresponding HR is a good estimate for relative workload. Taking the mean HR values of the 41 subjects in our study who had undergone HR_sh_ measurement, there was a slight exceedance (mean HR 100.2 b/min) of the standards of calculated mean HR value at 30 % VO_2,max_ (96.6 b/min); ns). Here, 66 % of the individuals had mean HR_sh_ values above 30 % VO_2max_. Frings-Dresen and Kemper 1995, under laboratory conditions, showed that 33–59 % of the subjects, depending on the waste collector activity (bags, different container volumes), exceeded the 30 % of VO2max.

Comparing these results with the oxygen uptake of the 13 individuals from the 1-h VO_2_ measurement, the means even exceeded the reference of 30 % VO_2,max_ significantly (mean VO_2,1h_ 1103 ml/min vs. calculated VO_2_ at 30 % VO_2,max_ of 737.3 ml/min, *p* < 0.05). All subjects achieved a mean VO_2_, which was above the reference limit of 30 % VO_2,max_, with a total range of 35–69 % VO_2,max_. These results are consistent with the relation between HR_1h_ and HR_sh_ as the 1-h values were slightly but significantly higher than HR_sh_. Nevertheless, in both specifications (HR and VO_2_), very high values have been found, which reflects the high continuous work load of refuse collectors.

In general, exercises that are performed with a HR_R_ > 30 % for an 8-h shift are assumed to be at high cardiovascular load (Ilmarinen et al. 1991; Shimaoka et al. 1998). With long-term HR measurement for a work shift of 6.7 h, 39 % of residual waste collectors, 33 % of organic waste collectors, and 39 % of the street cleaners had %HR_R,sh_ values that were higher than 30 % HR_R_. These findings are consistent with Kuijer et al. (1999), who found 36.4 %HR_R_ for refuse collectors and 22.6 %HR_R_ for street sweepers. Therefore, we can conclude that refuse collectors and street cleaners have high endurance performance and high cardiovascular load during work.

Åstrand et al. (2003) specified easy, moderate, and heavy work during an 8-h work shift on the basis of oxygen consumption at <600, 600–1000, and >1000 ml/min VO_2_, respectively, and required a maximum VO_2_ for work at 40 %VO_2,max_ at <1500, <1500–2500, and >2500 ml/min, respectively. When compared to Åstrand’s requirements of workload, the refuse collectors in our study had a mean VO_2,1h_ of 1103 ml/min during work corresponding to 46 % VO_2,max_ (Table 4) and a mean VO_2,max_ of 2623 ml/min during CPX corresponding to oxygen uptake under heavy physical work. This confirms Åstrand’s findings; the workload of refuse collectors can be classified in the upper field of heavy work. Whether the relatively high physical endurance is a health risk for the refuse collectors remains open. In our initial cross-sectional study, we found no evidence to this.

### Comparison with other occupations

Compared to jobs which are commonly referred to as physically heavy, the relative workload found in this study was rather high. The means for HR_sh_ and %HR_max_ (Table 3) during one work shift are consistent with Wultsch et al. (2012) findings for workers from waste processing (activities were not differentiated). They found mean HR_sh_ 100 b/min for male and 120 for female, 59 and 65 % HR_max_, respectively. Compared to the other investigated professions (workers in metal industry, slaughterhouse work, or healthcare business) referred in this study (Wultsch et al. 2012), our findings on the physical demand of refuse collectors were higher. Compared to a study with housekeepers which also used a portable spiroergometric system for field measurements (MJ Fröhlich, personal communication), we found similar values at HR_1h_ and VO_2,1h_ to those they determined with 112 b/min and 1.06 l/min, respectively. However, compared to portable spiroergometric measurements with lumberjacks (Hagen et al. 1993)—their job is considered to be the hardest form of physical work (with 49 % VO_2,max_ for the younger, 53 % VO_2,max_ for the older, and a HR_sh_ of 138 and 126 b/min, respectively)—our measurement results were rather low.

Other studies with refuse collectors have also reported similar HR values to those found in our study. Kemper et al. (1990) have found a mean HR_sh_ of 99.5 b/min in Dutch refuse collectors during one work shift, and—compared to the threshold value of 30 % VO_2,max_ calculated over HR_sh_—30 % of their participants had exceeded that limit. Furthermore, they also established a linear relationship between HR and VO_2_ during work, but they did not describe this correlation further. In a recent study with Brazilian refuse collectors, Anjos et al. (2007) outlined a mean HR for the total working time at 97.6 b/min, 53.4 % HR_max_, and 32.8 % HR_R_; nevertheless, their results were partially lower than those found in the present study. In addition, they identified HR values during the actual working time which can be compared with our values for 1 h of continuous work. A recent Japanese study by Tsujimura et al. (2012) found mean HR values for garbage collectors of 97.5 b/min, which were similar to the Brazilians but lower than our findings. These studies of refuse collectors, however, determined the workload only by the HR without VO_2_ field measurements.

## Conclusion

The present study demonstrates that HR and oxygen consumption are strongly correlated even during field measurements of the heavy dynamic work of the refuse collectors. Therefore, HR measurement is a valuable tool for evaluating the parameters of physiological workload during work. But the correlation between HR and VO_2_ was stronger under steady conditions in the laboratory, while HR can also be influenced by several external circumstances. In addition, we included only persons without heart disease or medication. In persons with cardiac disease or HR influencing medication, the sole determination of HR cannot replace the measurement of VO_2_. Therefore, if possible, the determination of VO_2_ should be aimed in the field measurement.

Refuse collectors exceed the upper limits set for physical work stress in the literature (Åstrand et al. 2003; Ilmarinen et al. 1991; Shimaoka et al. 1998). But all investigated employees were in our study within their individual reference limits of physical capacity and aerobic fitness, both in terms of absolute and relative HR as well as in oxygen consumption. The three task-specific groups (RWC, OWC, SC) did not differ in workload. The results of the present study can finally confirm the high workload of refuse collectors with the determination of VO_2_ at work. In addition, the endurance workload of refuse collectors is well above the hitherto recommended limits. The currently applicable limits for an 8-h shift with a maximum of 33–40 % of the individual’s VO_2 max_ or HR_R_ > 30 % should be reviewed. Other field measurements with determination of oxygen uptake with other physically hardworking professionals are necessary.
